# The role of imaging in acute pancreatitis

**DOI:** 10.1007/s11547-021-01359-3

**Published:** 2021-05-12

**Authors:** Maria Gabriella Brizi, Federica Perillo, Federico Cannone, Laura Tuzza, Riccardo Manfredi

**Affiliations:** Dipartimento di diagnostica per immagini, Radioterapia, Oncologia ed Ematologia, Fondazione Universitaria “A. Gemelli”, IRCCS - Università Cattolica del Sacro Cuore, Largo Agostino Gemelli, 8, 00168 Rome, Italy

**Keywords:** Acute pancreatitis, Computed tomography (CT), MRI, Magnetic resonance cholangiopancreatography (MRCP), Interstitial edematous pancreatitis, Necrotizing Pancreatitis

## Abstract

Acute pancreatitis is one of the most commonly encountered etiologies in the emergency setting, with a broad spectrum of findings that varies in severity from mild interstitial pancreas to severe forms with significant local and systemic complications that are associated with a substantial degree of morbidity and mortality. In this article the radiological aspect of the terminology and classification of acute pancreatitis are reviewed. The roles of ultrasound, computed tomography, and magnetic resonance imaging in the diagnosis and evaluation of acute pancreatitis and its complications are discussed. The authors present a practical image-rich guide, applying the revised Atlanta classification system, with the goal of facilitating radiologists to write a correct report, and reinforcing the radiologist’s role as a key member of a multidisciplinary team in treating patients with acute pancreatitis. Computed tomography is the most performed imaging test for acute pancreatitis. Nevertheless, MRI is useful in many specific situations, due to its superiority soft tissue contrast resolution and better assessment of biliary and pancreatic duct, for example in the ductal disconnection. The purpose if this article is to review recent advances in imaging acquisition and analytic techniques in the evaluation of AP.

## Definition

Acute pancreatitis (AP), an inflammatory disorder of the pancreas, refers to the autodigestion of the pancreas, in which pancreatic enzymes injure pancreatic tissue and lead to dysfunction of the gland, as well as remote organs and systems. The epidemiology of diseases often changes with time—for pancreatitis, this aspect is certainly true. The reasons for such changes are many: population growth and migration, change in patterns of alcohol consumption and tobacco smoking, rising rates of obesity and recognition of metabolic causes of pancreatitis, and increasing use and improving quality of imaging modalities [1–3].

## Epidemiology

### Incidence

The global pooled incidence of AP is 34 cases per 100,000 general population per year [95% confidence interval (CI) 23–49], with no statistically significant difference between men and women [4]. The disease predominantly affects people between 60 and 75 years old [5]. Also, we can identify regions with high incidence (that are, those with incidence more than 34 cases per 100,000 general population per year) are the North America and Western Pacific regions (as defined by the WHO).

Recurrent AP developed in 21% (95% Cl 17–26%) of patients after the first episode of AP, and chronic pancreatitis developed in 36% (95% Cl 20–53%) of patients after recurrent acute pancreatitis [1].

### Prevalence

The notion of prevalence is typically considered in the context of chronic diseases, yet the prevalence of acute conditions can also be of importance [1]. The pancreatologists had not focused their attention on estimating the prevalence of AP, because it was believed that the majority of patients do not develop long-term consequences, while data suggest that even patients with mild AP (around 80%) have at least twofold higher long-term risk of diabetes mellitus than people in the general population [6, 7]. Thus, a knowledge of prevalence might enable quantification of the predicted burden of sequelae attributable to acute pancreatitis in the general population and guide the effective allocation of health care resources [1].

### Mortality

The pooled mortality from an episode of AP in seven population-based cohort studies evaluated in the systematic review by Xiao et al. [4] was 1.16 (95% CI 0.85–1.58) per 100,000 general population per year. Determinants for increased risk for mortality in AP are well-established and include persistent organ failure and infected pancreatic necrosis [8–10]. There are two peaks of lethality in AP: the first one, connected with early dysfunction of organs, begins after one week from the disease onset; the second peak, connected with infected centre’s of necrosis, onsets from the second week of the disease.

## Clinical presentation

AP is an inflammatory condition of the pancreas that can cause local injury, systemic inflammatory response syndrome, and organ failure; worldwide AP is a common condition associated with substantial suffering, morbidity, and cost to the health care system [11]. According to the revised Atlanta classification, accurate diagnosis of AP requires at least two of the following three diagnostic features [12]:

1. Abdominal pain consistent with AP.
2. Serum lipase or amylase levels that are at least 3 times the upper limit of the normal range, and
3. Findings of AP on cross-sectional imaging (computed tomography—CT—or magnetic resonance imaging—MRI).

If abdominal pain suggests strongly that AP is present, but the serum amylase and/or lipase activity is less than three times the upper limit of normal, as may be the case with delayed presentation, imaging will be required to confirm the diagnosis [13, 14]. If the diagnosis of AP is established by abdominal pain and by increases in the serum pancreatic enzyme activities, a CT is not usually required for diagnosis in the emergency room or on admission to the hospital. The onset of the pancreatitis is considered to coincide with the 1st day of pain, not the day on which the patient presents for care or the day of hospital admission [15].

## Phases of AP

AP is divided into early and late phases.

- The early phase—first week after the onset—is characterized by activation of the cytokine cascade with resultant systemic inflammatory response syndrome (SIRS). If SIRS persists there is an increased risk of developing organ failure, that can be—transient—if it resolves within 48 h or—persistent—if it persists for > 48 h [16–18].
- The late phase, starting in the 2nd week and can lasts for weeks to months, occurs only in patients with moderately severe or severe pancreatitis, as defined by persistent organ failure and by local complications [12] and it is characterized by the presence of local complications, systemic manifestations and/or by transient or persistent organ failure.

## Grading of AP

According to the revised Atlanta classification, the severity of AP identifies three classes:

- Mild AP, with no organ failure, and no local or systemic complications. Patients with mild AP generally do not require pancreatic imaging, and mortality is very rare [19].
- Moderately severe AP characterized by the presence of transient organ failure or local or systemic complications in the absence of persistent organ failure.
- Severe AP, characterized by persistent organ failure, that may be single or multiple organ failure; these patients can have one or more local complications, and have an increased risk of death [16–18].

To correlate complications and mortality several clinical scoring systems like Marshal or APACHE (Acute Physiology and Chronic Health Disease Classification System) were designed [12]. Balthazar et al. in 1990 introduced instead the CT severity index for assessment of AP. In 2004 Mortele et al. [20] introduced the MCTSI, which includes as prognostic indicators the pancreatic inflammation, the pancreatic necrosis and extrapancreatic complications (Table 1).

**Table 1:** Modified CT severity index^20^

Prognostic indicators	Points
*Pancreatic inflammation*	
Normal pancreas	0
Intrinsic pancreatic abnormalities with ot without inflammatory changes in pancreatic fat	2
Pancreatic or peripancreatic fluid collection or peripancreatic fat necrosis	4
*Pancreatic necrosis*	
None	0
≤ 30%	2
≥ 30&	4
*Extrapancreatic complications*	
One or more of pleural effusion, ascites, vascular complications, parenchymal complications, or gastrointestinal tract involvement	2

## IEP versus necrotizing pancreatitis

AP can be subdivided into two types, according to the pathologic changes: interstitial edematous pancreatitis (IEP) and necrotizing pancreatitis [12].

**Fig. 1:**
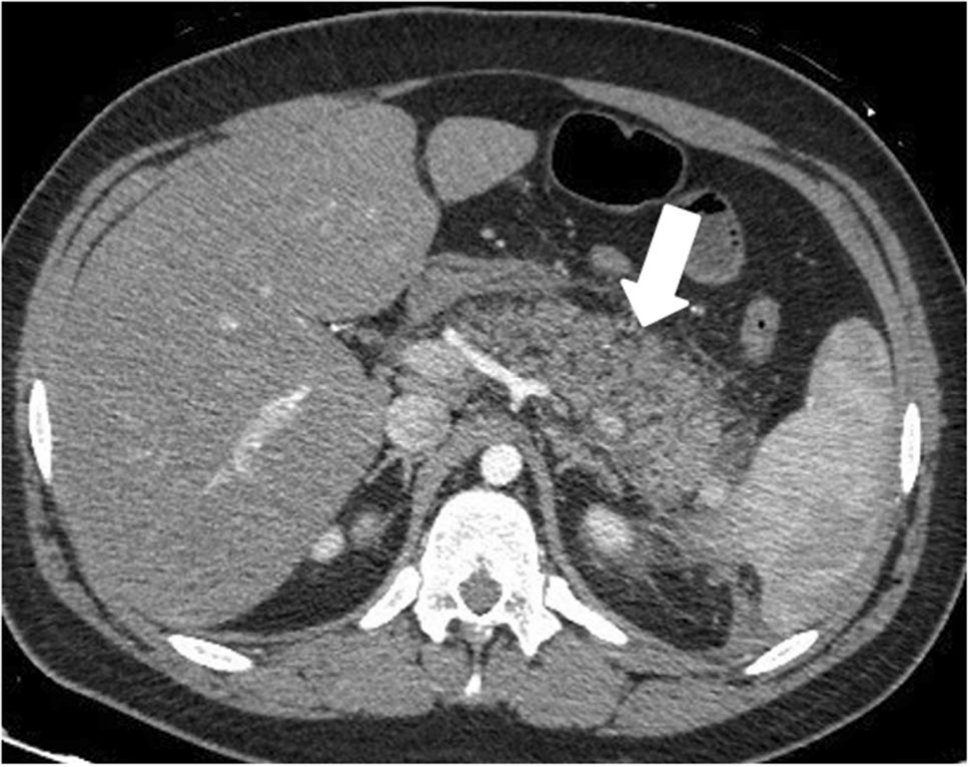
Interstistial acute pancreatitis in 17-year-old man, after cannabinoids abuse: axial IV contrast-enhanced CT scan shows mild diffuse enlargement of the whole pancreatic gland with poorly defined contours (arrow); the enhancement of the pancreatic parenchyma is normal and there are no foci of glandular necrosis

**Fig. 2:**
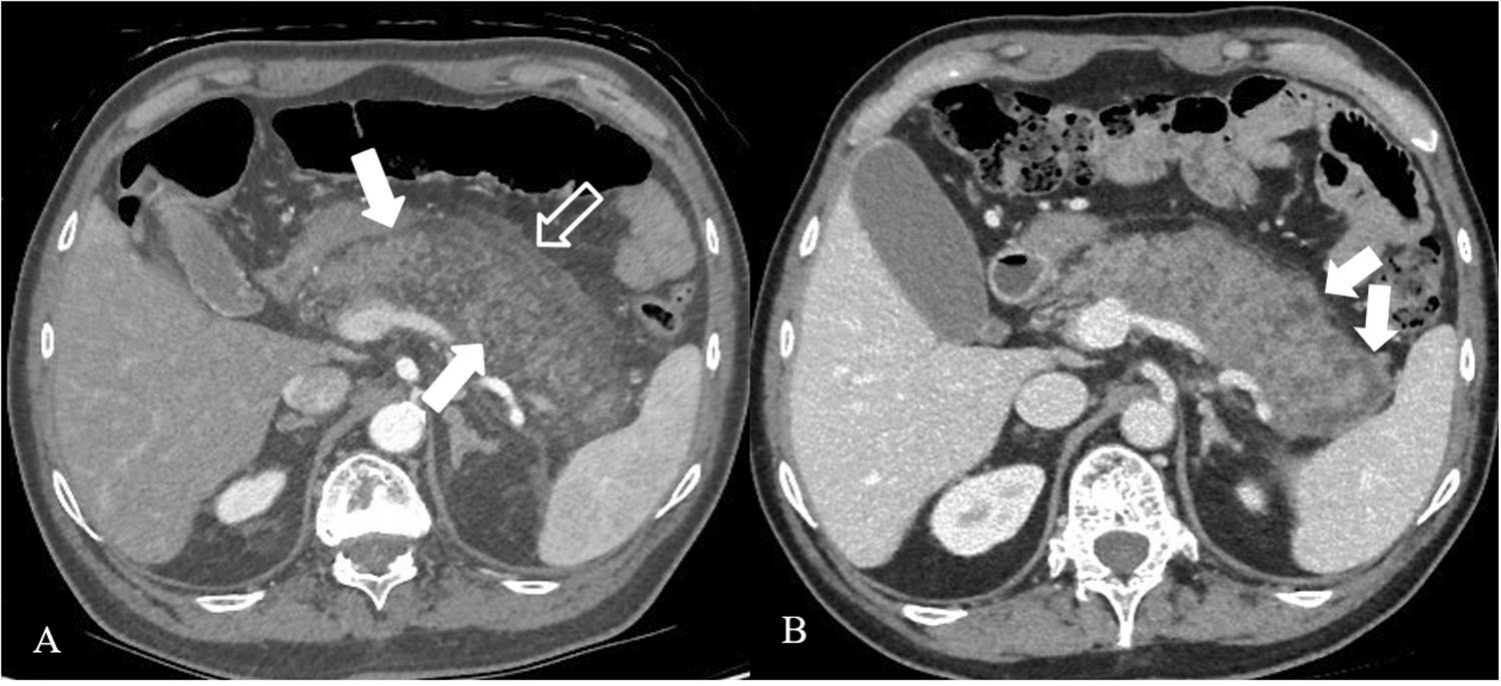
Acute pancreatitis in a 61 years-old man. The axial contrast-enhanced CT scan after 3 days from the onset (**a**), in pancreatic arterial phase, shows edematous pancreas (arrows), with disomogeneous and reduced enhancement, with no necrotic area in the contest. It is also evident mild peripancreatic fatty stranding (empty arrow). TC study after nine days of the onset (**b**) shows heterogeneous contrast enhancement of the pancreatic parenchyma, with focal hypovascular areas due to the necrosis (arrows)

IEP is more common and represents non necrotizing inflammation of the pancreas. Most patients, above 69%, have diffuse enlargement of the pancreas, occasionally it is localized, due to inflammatory edema. On contrast-enhanced Computed Tomography (CECT), the pancreatic parenchyma shows relatively homogeneous enhancement, but there are not unenhanced (necrotic) areas (Fig. 1). The peripancreatic fat usually shows some inflammatory changes of haziness or mild stranding; there may also be some peripancreatic fluid [12] (Fig. 2a); the clinical symptoms usually resolve within the 1st week.

Five—10%—of patients with acute pancreatitis develop a necrotizing pancreatitis. The necrosis may involve either the pancreatic parenchyma and/or the peripancreatic tissues. There are three subtypes of necrotizing pancreatitis, based on the anatomic area of necrotic involvement:

- Pancreatic only
- Peripancreatic only
- Combined pancreatic and peripancreatic [15].

**Fig. 3:**
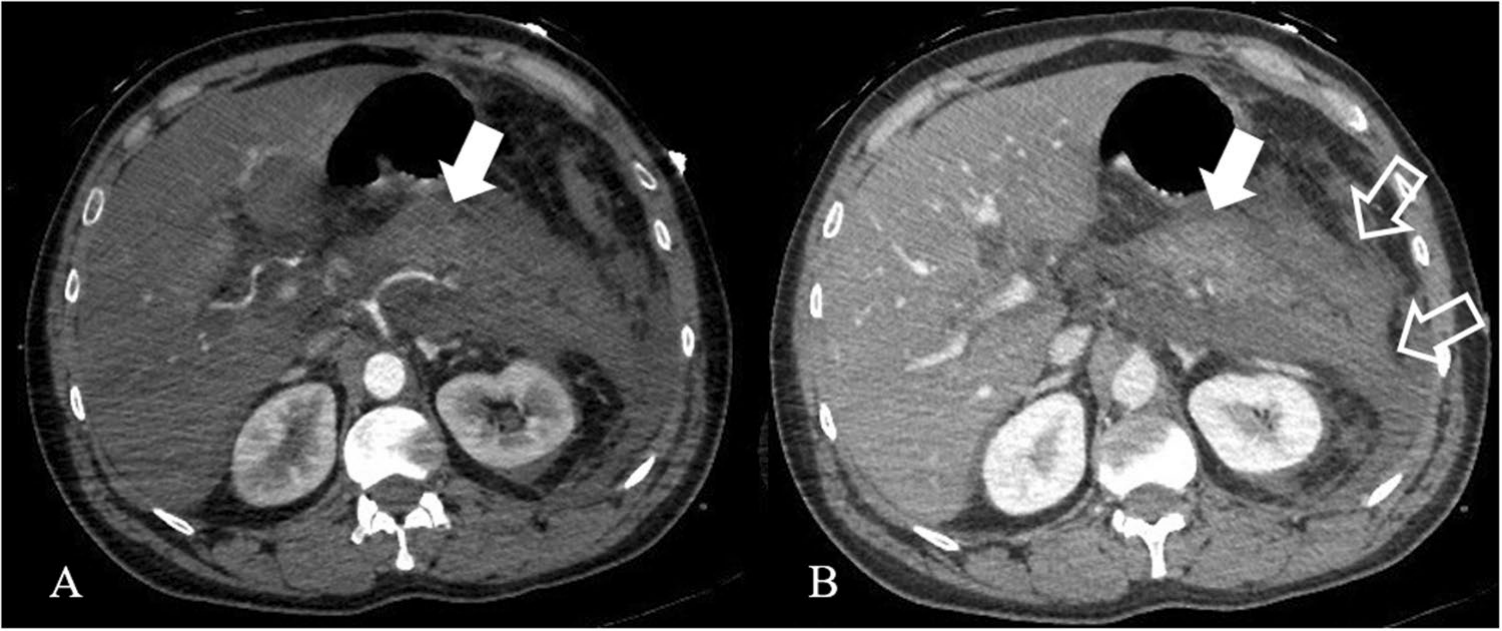
Necrotizing Pancreatitis in a 39 years-old man, with acute abdominal pain and sepsis. Axial contrast-enhanced CT scans during the arterial phase (**a**) and portal phase (**b**) show enlarged pancreas with poorly defined contours and decreased enhancement of the pancreatic parenchyma (arrows), surrounded by heterogeneous fluid collection (empty arrows in **b**)

Necrotizing pancreatitis most commonly manifests as necrosis involving both the pancreas and peripancreatic tissues in the 75% of the cases, while less commonly—in the 20%—it manifests as necrosis of only the peripancreatic tissue, and in 5% as necrosis of the pancreatic parenchyma alone [12]. The combined subtype demonstrates non-enhancing pancreatic parenchyma, as well as non-enhancing heterogeneous peripancreatic collections, and typically accumulating in the lesser sac and anterior pararenal space (Fig. 3). Peripancreatic necrosis alone occurs in 20% of cases, with normal enhancement of the pancreas, while in the peripancreatic tissues there is necrosis, with collections. Pancreatic necrosis alone (Fig. 2b) is the least common subtype, occurring in 5% of cases [15].

An early CECT may underestimate the extent of pancreatic and peripancreatic necrosis because the impairment of pancreatic perfusion and signs of peripancreatic necrosis evolve over 7 days, therefore a CT examination should not be performed before 72 h, from the onset of symptoms, in order to grade the severity of the disease [12, 19, 21].

In the first few days of the illness, the pattern of perfusion of the pancreatic parenchyma as seen on CECT may be patchy, with variable attenuation before the area of impaired enhancement becomes more demarcated and/or confluent. In these cases, repeat CECT 5–6 days later is more accurate for the diagnosis of necrotizing pancreatitis [12] (Fig. 2b).

**Fig. 4:**
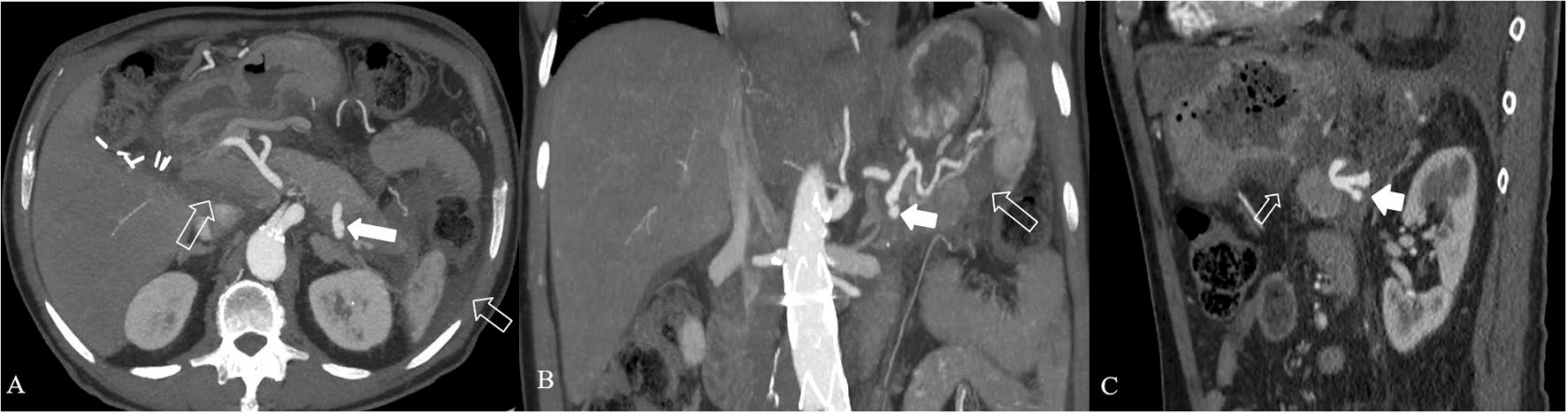
Splenic artery pseudoaneurism in a 75 years-old man with RAP. The contrast-enhanced CT scans in the arterial phase axial MIP (**a**), coronal MIP MPR (**b**) and sagittal MPR (**c**) show splenic artery pseudoaneurism (white arrows), in a necrotizing pancreatitis with evident peri-pancreatic collection (empty arrows) extending up to anterior pararenal space of the retroperitoneum, gastrohepatic and gastrosplenic

Imaging protocol varies by institution. CECT is usually performed employing a protocol depending on the clinical question; at our institution protocol includes an unenhanced scan, followed by arterial phase and portal venous phase. Unenhanced images may help to depict free intrabdominal fluid, for discriminating active bleeding from metal clips and calcification. The arterial phase on the upper abdomen is performed at 35–40 s after the initiation of IV contrast or 15–20 s after the peak enhancement, the so-called pancreatic phase with maximum contrast enhancement of the pancreatic parenchyma and then the portal venous phase from the top of the diaphragm, including the entire abdomen. Iodinated intravenous contrast is essential to evaluate of pancreatic necrosis, as well as evaluate for vascular complications such as pseudoaneurysm or splenic thrombosis (Fig. 4).

## Pancreatic and peripancreatic collections

The revised Atlanta classification distinguishes different kinds of collections according to if they are purely fluid collections or containing necrotic debris in addiction to fluid and considering the time course (≤ 4 weeks or > 4 weeks from the onset of the pain).

**Fig. 5:**
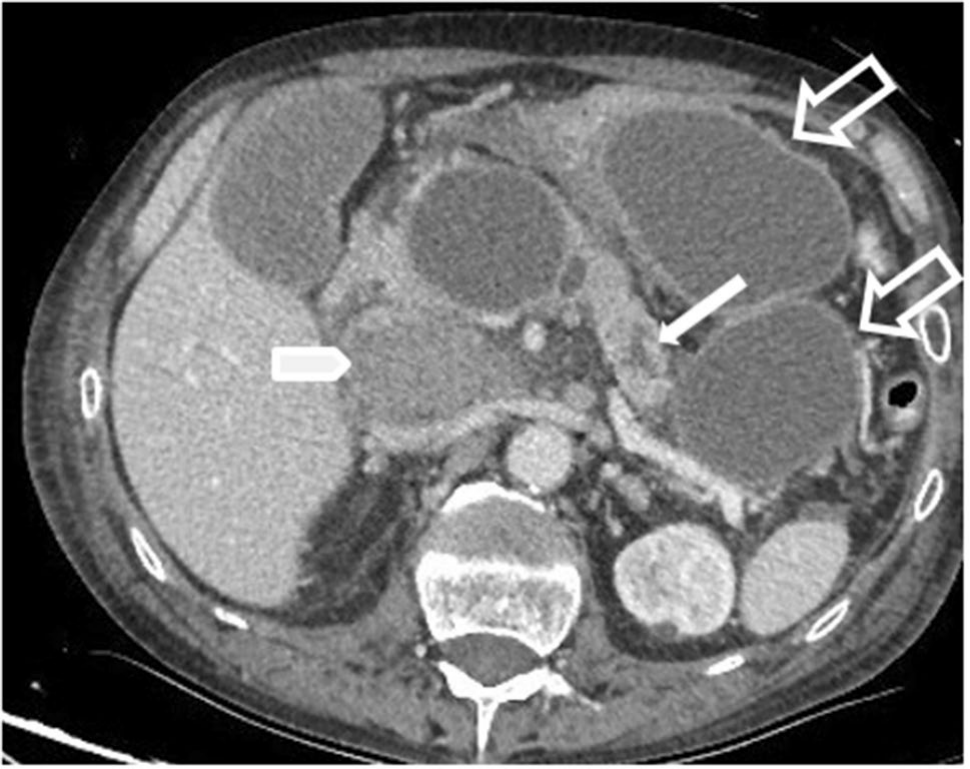
Pseudocyst: 61 years old man, with history of alcohol abuse and hospitalized for necrotizing pancreatitis. Contrast-enhanced CT scan after 4 weeks from the onset, show a hyperdense collection in the retroperitoneum, behind the head of the pancreas and the duodenum (arrow head) suggesting previous bleeding. No signs of active bleeding were evident. Other pseudocysts are localized in the peri-pancreatic fat, between body and tail (empty arrows). Wirsung duct is also dilated (thin arrow)

In patients with IEP in the first 4 weeks acute peripancreatic fluid collections (**APFCs**) can occur, that are fluid collections in the peripancreatic region, with no well-defined walls and no internal solid components (Fig. 1). Most APFCs remain sterile and usually resolve spontaneously without intervention; on CECT they appear as homogeneous collections, with low attenuation, frequently seen in the lesser sac and in the anterior pararenal space. If an APFCs has not resolved after 4 weeks, it becomes more organized and develops a capsule that manifests as an enhancing wall at CECT, containing only fluid, no necrosis. At this point the collection refers to as a **pseudocyst**, a well-circumscribed peripancreatic fluid collection, surrounded by a well-defined enhancing capsule (fibrous or granulation tissue) (Fig. 5). They generally resolve spontaneously, while the 50% of persistent pseudocysts will cause clinical symptoms or complications, which can include secondary infection, pain, hemorrhage secondary to erosion into adjacent vessels, decompression or rupture, or local mass effect (Table 2).

**Table 2:** Pancreatic and peripancreatic collections in IEP^12^

Collection	Time after onset of pain (week)	Location	Imaging features CECT	Imaging features MRI
APFC	≤ 4	Extra -pancreatic	Homogeneous collection with fluid density Confined by normal peripancreatic fascial planes No definable wall Adjacent to pancreas (no intrapancreatic extension)	Homogeneously hypointense on T1WIs and hyperintense on T2WI No cystic walls and no solid components
Pseudo-cyst	≥ 4	Extra-pancreatic	Well defined wall Usually round or oval Homogeneous fluid density No non-liquid component	Thin smooth wall Homogeneous intensity on T1WIs and T2WIs of the liquid content No solid component or debris in the fluid

**Fig. 6:**
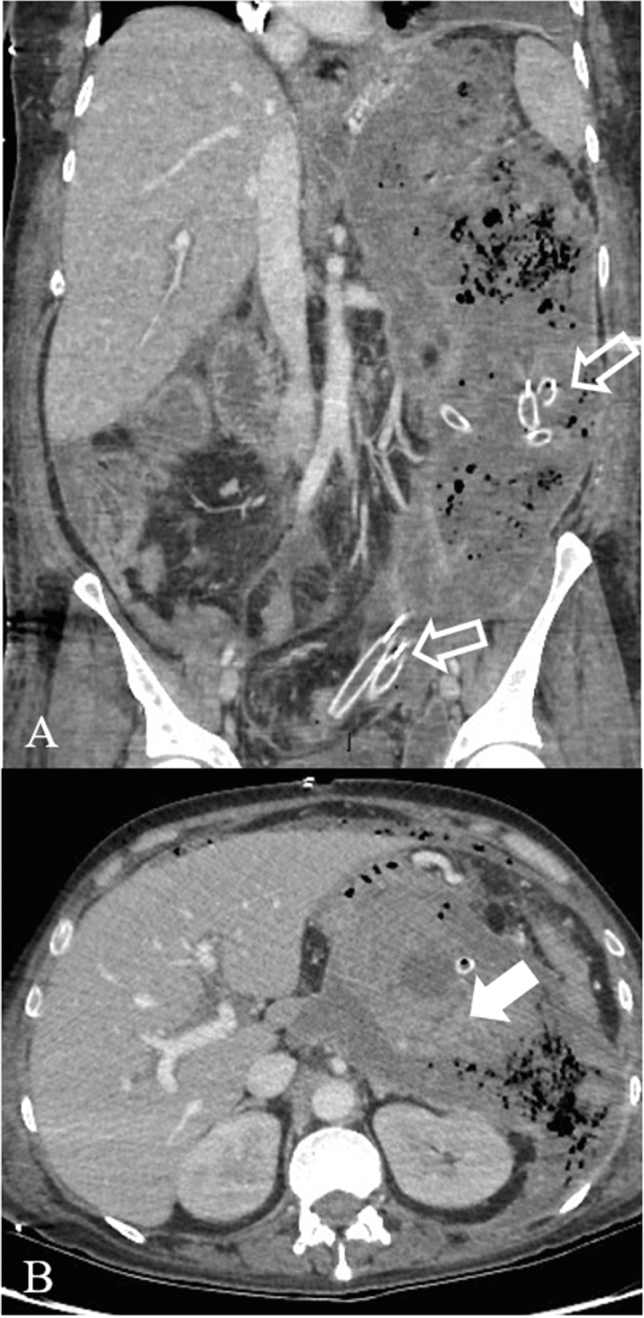
Necrotizing Pancreatitis. The same case as the previous in Fig. 3, after 1 month of the onset of the symptoms, and 1 week after surgical necrosectomy, the contrast-enhanced CT scans in the portal phase show a peri-pancreatic collection with bubbles air and drainage tubes in the collection (empty arrows). Coronal MPR image (**a**) shows the extention of the collection. Axial scan (**b**) show diffuse alterations of the CE of the parenchyma, due to necrosis (arrow)

**Fig. 7:**
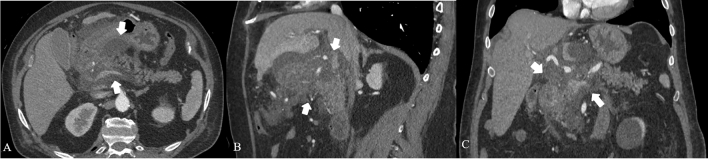
ANCs in biliary AP in a 74 years-old man. The contrast-enhanced CT axial scan (**a**) shows the presence of an acute necrotic fluid collection (ANCs) interesting the head and the body of the pancreatic gland (white arrows), extending in the fat surrounding the gland up to the gastrohepatic ligament. The sagittal MPR (**b**) and coronal (**c**) better show the extention of the collection

**Fig. 8:**
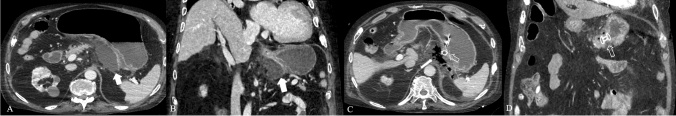
Necrotizing AP in a 74 years-old man with a history of AP, 3 weeks after the onset. The contrast-enhanced CT axial scan (**a**) and the coronal MPR (**b**) show the presence of collection in the lesser sac up to the lesser curvature of the stomach (white arrows). The contrast-enhanced CT axial scan (**c**) and the coronal MPR (**d**) 5 days after the previous examination show the drainage of the collection by cystogastrostomy (empy arrow), and the presence of gas-collection (thin arrow in **c**)

**Fig. 9:**
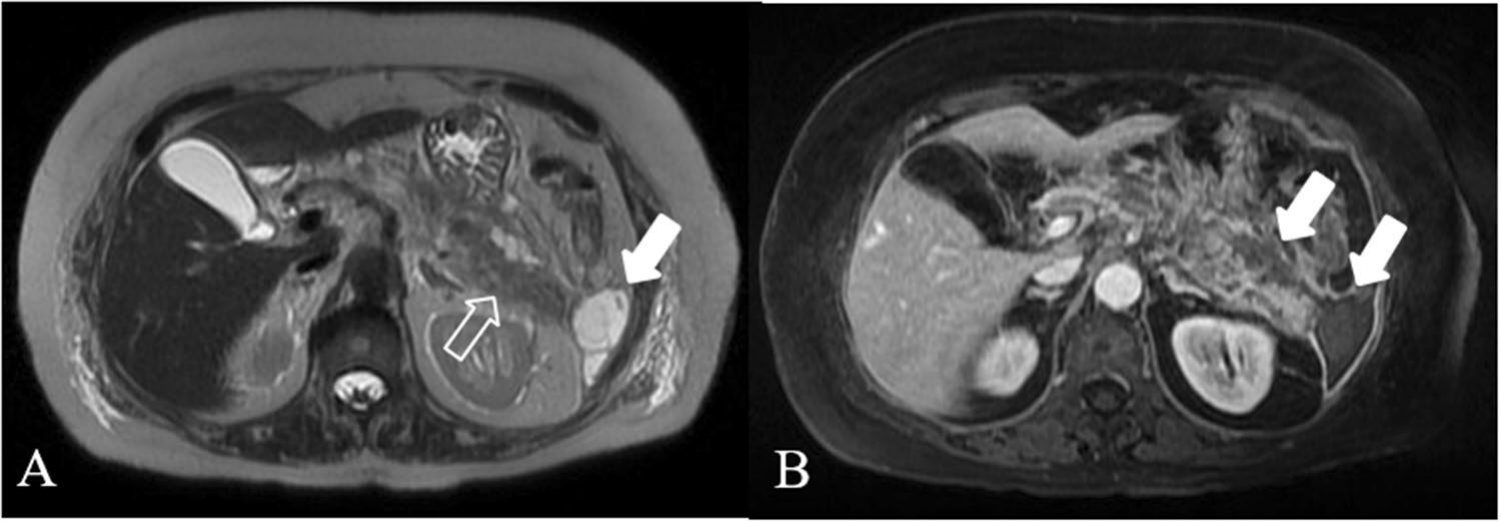
Walled off necrosis. 73 years old woman undergoing statins and amiodarone treatment. T2 weighted MR image (**a**) and post-gadolinium T1w scan (**b**), show the presence of a heterogeneous encapsulated fluid collection suggesting walled-off necrosis (WON); the WON extends in the pancreatic and peripancreatic area, up to the left anterior pararenal space and left paracolic space (arrows). Diffuse hyperintensity on T2w images of the pancreatic parenchyma indicates the presence of parenchymal edema (empty arrow in **a**)

**Fig. 10:**
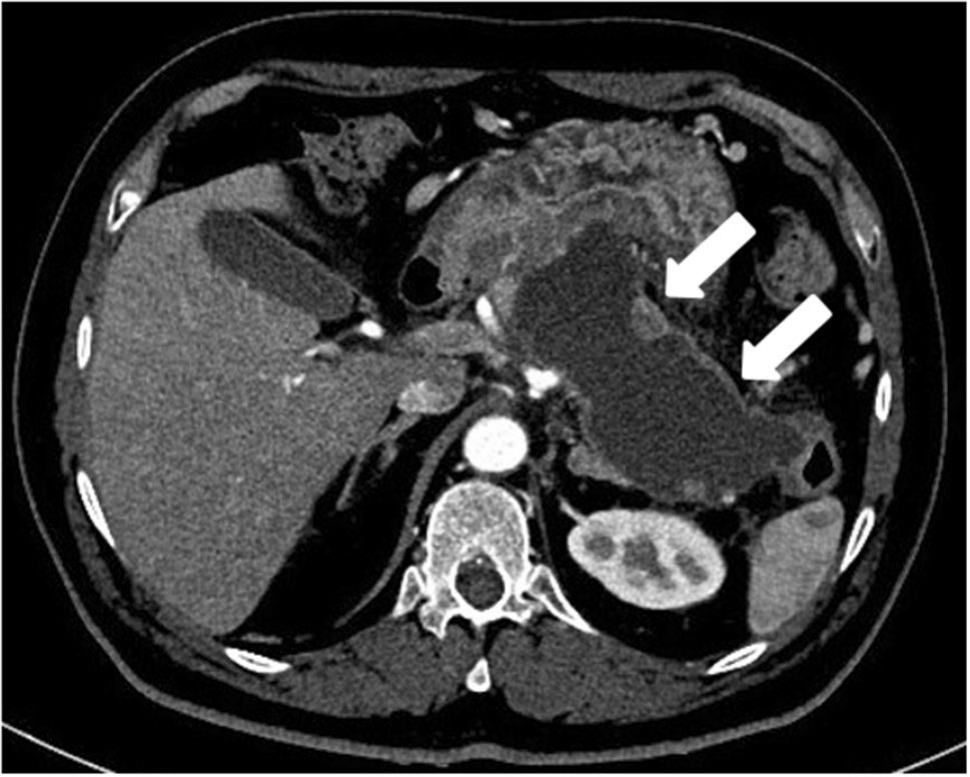
WON evolution. Acute necrotizing pancreatitis in a 61-years-old man. Four weeks after the onset axial CECT scan shows the presence of an encapsulated collection (WON) in the peripancreatic fat near the body and the tail of the gland (arrows)

Acute necrotic collection (**ACNs**) present within the first 4 weeks of necrotising pancretitis and are poorly organized necrotic collections (Figs. 3, 6, 7, 8a–b). On CECT, ACNs are heterogeneous in appearance and have no definable wall enclosing the collection; however even if the collection is homogenous, it is considered ANC when associated with known pancreatic parenchymal necrosis. On unenhanced CT, the presence of fat attenuation within a pancreatic collection is helpful at identifying necrosis and can also help differentiate between ANCs and APFCs [12, 15]. After 4 weeks of necrotising pancreatitis ACNs become **WON** (walled off necrosis). It resembles a pseudocyst, but it can be differentiated on CECT by the presence of internal solid components (Figs. 9, 10). As ACNs, WON may be intra- or extra-pancreatic [12]. When WONs develop in the setting of a normally enhancing pancreas on CECT, a T2-weighted MRI or ultrasound may be necessary to help to identify the presence of debris in the fluid collection to distinguish WON from pseudocyst [22, 23] (Table 3).

**Table 3:** Pancreatic and peripancreatic collections in necrotizing pancreatitis^12^

Collection	Time after onset of pain (week)	Location	Imaging features CECT	Imaging features MRI
ANC	≤ 4	Intra- and/or extra-pancreatic	Heterogeneous and non-liquid density of varying degrees in different locations No definable wall encapsulating the collection	Mixed signals on T1WI and T2WI Necrotic tissue fragments in the collection
WON	≥ 4	Intra- and/or extra-pancreatic	Encapsulated collection of pancreatic and/or peri-pancreatic necrosis Heterogeneous Well defined wall	Encapsulated effusion contains non-liquid substances, flocculent, with banded tissue fragments, free and floating

## Complications

Any collection can be sterile or infected, although infection occurs more frequently in necrotic collections. Infection should be clinically suspected since the only imaging finding of an infected collection is the presence of gas within the collection. Wall enhancement is not a reliable indicator of infection since it is invariably present in mature collections (pseudocysts and WONs). The gas often appears as multiple small bubbles scattered throughout the collection owing to the complex nature of necrotic collections [12]. Infected collections can also manifest with gas bubbles due to a pancreatic-enteric fistula, which can occasionally be seen when necrotic collections erode through the bowel wall, most commonly in the colon and the duodenum [24].

Patients with necrotizing pancreatitis require an individualized multidisciplinary management approach to reduce mortality and prevent associated complications. The nowadays accepted management is the “step-up” approach that aims to utilize the least invasive technique first, with progressive escalation for treatment failure [25]. Current evidence-based treatment includes an initial step of drainage (either percutaneous catheter or transluminal endoscopic—Fig. 8c–d) and then frequent re-evaluation of the clinical success of this approach. Surgical or endoscopic transluminal debridement is now only required with lack of clinical resolution and is delayed until necrosis has become WON [26].

**Fig. 11:**
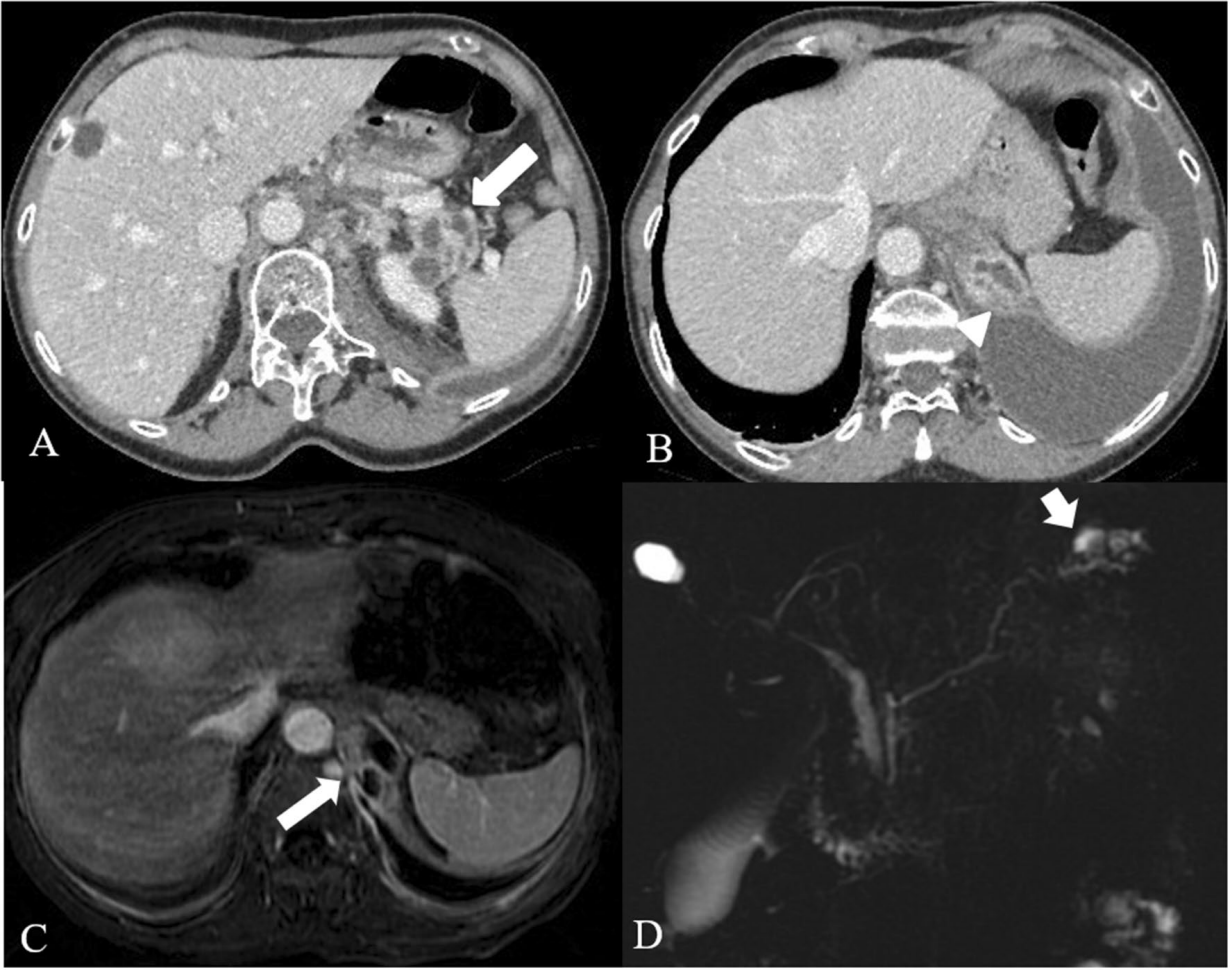
Fistula with pleural cavity in a 66 years-old woman with acute necrotizing pancreatitis. Multiple encapsulated fluid collections in the tail of the pancreatic parenchyma (arrow in** a**), as for necrotic spots. These collections extend upt to the left hemidiaphragm with pleural fistula and pleural effusion (arrowhead in **b**). Axial (**c**) post contrast T1w MR images and MRCP (**d**) confirm the pleural fistula (arrows)

Apart from collections, other complications may occur, such as vascular complications. Splenic vein thrombosis represents the most common vascular complication in patients with AP. The release of pancreatic enzymes in AP results in erosion of local vasculature which may lead pseudoaneurysm malformation as well as spontaneous hemorrhage; the most common source of bleeding is the splenic artery, portal vein, and other peripancreatic vessels [27]. Necrosis of the central pancreas results in the disruption of the main pancreatic duct in 40% of cases, that can be confirmed with pancreatic MRI and MRCP (Magnetic Resonance Cholangiopancreatography) (Fig. 11). Other complications of necrotizing pancreatitis are pancreatic duct strictures (Fig. 2), which may develop secondary to inflammation or healing following successful drainage of necrotic collections [27].

## Role of MRI in AP

In the emergency setting CT and ultrasound are the imaging modalities of choice because of accessibility, speed, and lower cost. Indeed, in the early phase of AP a CECT study, at 5–7 days after admission, is generally performed. In the late phase of moderately severe or severe acute pancreatitis, local complications evolve completely, such as the presence of infection within areas of necrosis. Since different local complications may require a variety of interventions, it is important to distinguish between them. For these reasons MRI has its own rule, more in the late phase, having a superior soft tissue contrast resolution and allowing better assessment of biliary and pancreatic ducts. MRI also does not use ionizing radiation, but it requires patient co-operation.

**Fig. 12:**
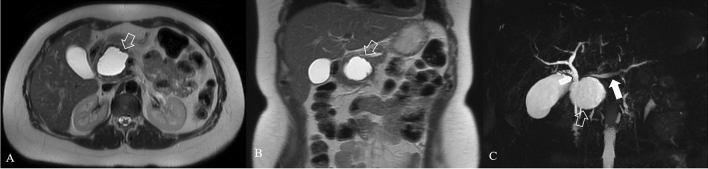
Pseudocyst in a 36 years-old woman, with AP, 12 weeks from the onset. Axial scan (**a**) and coronal scan (**b**) show the hyperintense collection in T2 weighted image (empty arrow) with dilated pancreatic duct (white arrow). MRCP (**c**) shows bile duct dilatation (arrowhead), due to the compression from the collection

Normal pancreatic anatomy is best depicted on T1 weighted fat-suppressed images, in which the pancreas is typically hyperintense because of pancreatic acinar proteins [28, 29]; on T2 weighted images, pancreatic parenchyma is typically hypointense. In necrotizing pancreatitis, the necrotic area displays hypointensity on T1WI, hyperintensity on T2WI and no enhancement after an injection of contrast agent such as Gd-DTPA [30]; if the necrotic zones on MRI contain gas may indicate infection [22]. On MRI scans acute peripancreatic fluid collections (**APFCs**) are homogeneously hypointense on T1WIs and hyperintense on T2WI, with no cystic walls and no solid components. On MRI pancreatic **pseudocysts** have a thin smooth wall, homogeneous intensity on T1WIs and T2WIs of the liquid content, and no solid component or debris in the fluid (Fig. 12). Pancreatic juice constantly overflows from the ruptured pancreatic duct, leading to a gradual enlargement of the cysts; for surgical indications it is important to visualize where the pancreatic duct breaks and its extent of rupture [31], for this reason T2WI, and also MRCP and multiplanar reconstruction are generally performed. MR with MRCP may be helpful in treatment planning of pancreatic pseudocysts by assessing the internal content and by displaying the relationship with the stomach or the duodenum to plan pseudocystostomy.

**Fig. 13:**
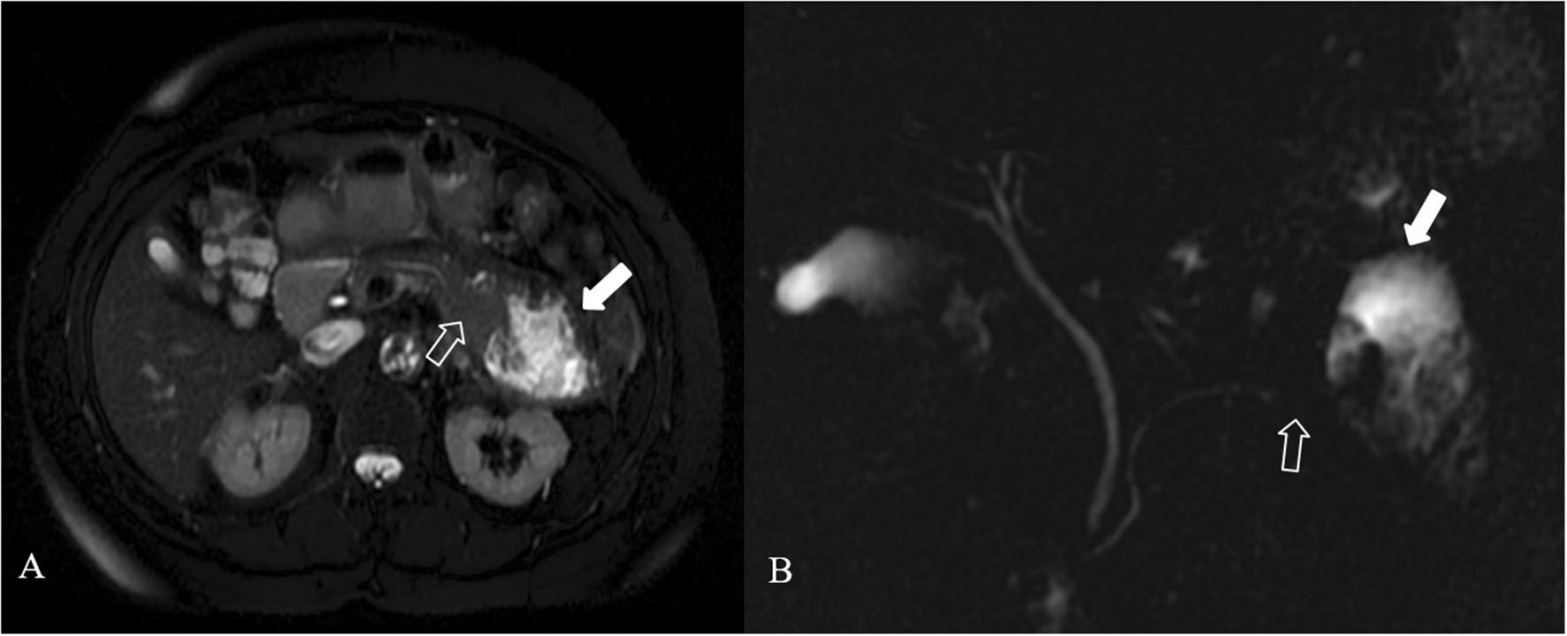
ACNs in a 72 years-old woman. T2 weighted MR image (**a**) and MRCP (**b**) 2 weeks after the onset show a heterogeneous collection in the pancreatic tail (white arrow), where there is a focal interruption of the Wirsung duct (empty arrow)

**ANCs** have no capsules, and they show mixed signals on T1WI and T2WI (Fig. 13); although MRI is sensitive in differentiating different components in the accumulation of peripancreatic tissue, when the necrotic tissue fragments are small, MRI cannot determine whether the liquid that appears with high signal on the liquid-suppressed T2WI is entirely inflammatory fluid without doping necrotic cells, and thus, MRI still cannot accurately determine ANCs [32]. The maturation stage of ANCs is the **WON** that on MRI scans appear as encapsulated effusion contains non-liquid substances, flocculent, with banded tissue fragments, free and floating, and there is no enhanced signal on the enhanced scans (Figs. 9b; 10); patients with WON are commonly complicated with infection.

**Fig. 14:**
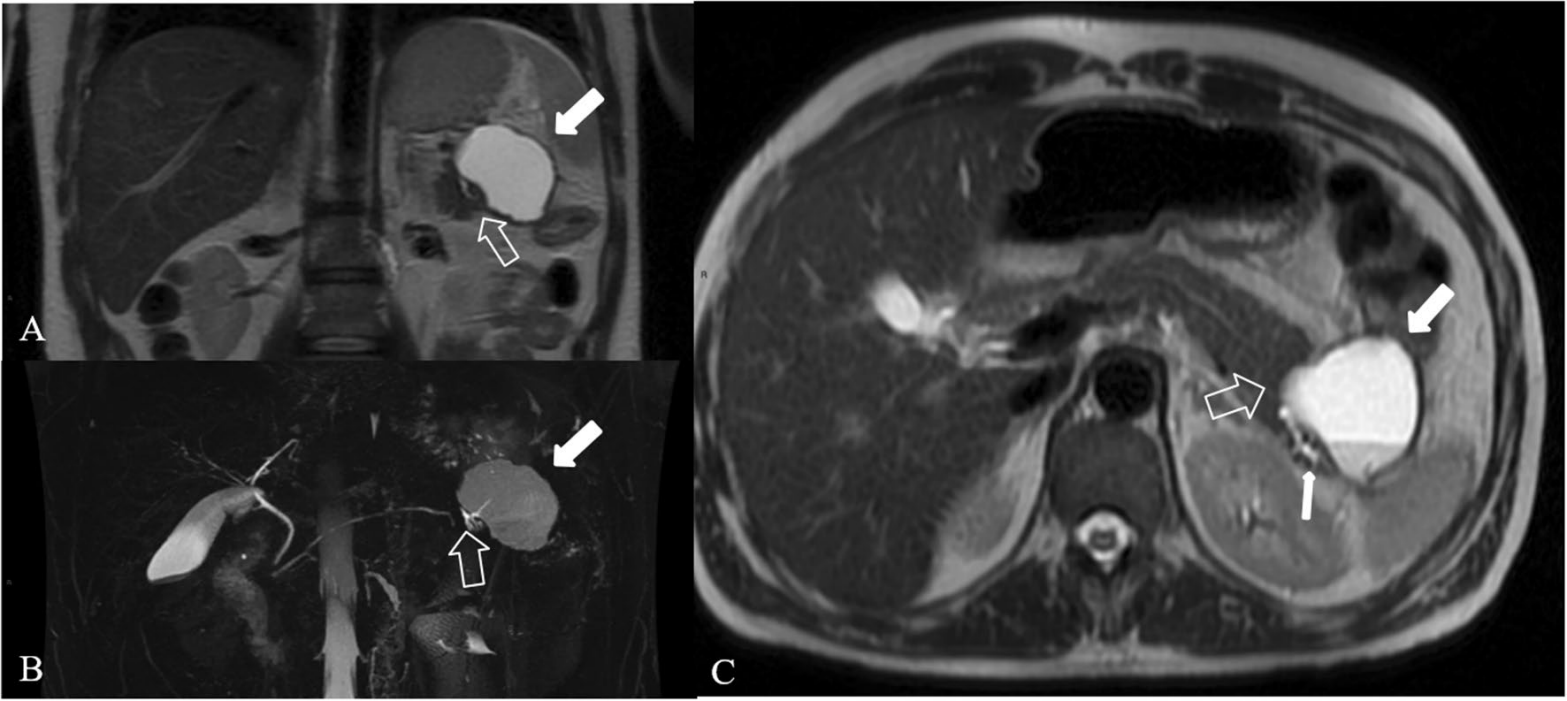
Disconnected pancreatic duct syndrome in a 41 years-old man. Coronal (**a**), axial (**c**) T2 weighted MR images and MRCP (**b**) 3 weeks after the onset of the symptoms show focal interruption of the Wirsung duct (empty arrow)—upstream pancreatic duct dilatation (thin arrow in **c**)—and a heterogeneous encapsulated fluid collection in the pancreatic tail (white arrow)

The severe necrotic pancreatitis with pancreatic duct disruption, called **disconnected pancreatic duct syndrome** (DPDS) (Fig. 14), has 10% to 31% as prevalence [33]. In these cases, the combination of MRI and MRCP provides a noninvasive method showing not only the pancreas and peripancreatic changes but also analyzing the proximal and distal ends of the ruptured main pancreatic duct and possible fistula. Therefore, the use of MRI and MRCP is more advantageous to evaluate the main disconnected pancreatic duct syndrome caused by acute necrotizing pancreatitis [34]. As the severity increases, the incidence of pancreatic duct rupture also increases. The diagnosis of pancreatic duct disruption should be considered if there is a peripancreatic necrosis area at least 2 cm, and MRCP shows that the main pancreatic duct of the upstream pancreatic tissue travels to the WON area of the intra and/or extra pancreatic tissue. MRCP performed with secretin is emerging as the imaging study of choice for the diagnosis of a disconnected pancreatic duct, which demonstrates a cutoff of the downstream pancreatic duct with enhancing upstream pancreatic parenchyma [35]; S-MRCP may on occasion shows the passage of exocrine output into the collection.

MRI is also sensitive to visualizing hemorrhages, which are hyperintense on T1WI during acute phase and have signals that persist longer than on CT images [36].

MRI can have a role in the identification of the etiology (alcohol and choledocholithiasis), especially MRCP in biliary microlithiasis, it is superior to CT in showing small ductal calculi, evaluating the pancreatic duct and biliary tree, demonstrating high sensitivity and specificity, and adding benefit of potentially obviating the more invasive ERCP [37–40].

## Recurrent acute pancreatitis (RAP)

After therapy, most patients with AP recover completely, but some of them can have other new episode and are said to have recurrent acute pancreatitis. RAP has been defined as a syndrome of multiple distinct acute inflammatory responses originating within the pancreas in individuals with genetic, environmental, traumatic, morphologic, metabolic, biologic, and/or other risk factors who experienced 2 or more episodes of documented AP, separated by at least 3 months.

Idiopathic recurrent acute pancreatitis is defined as RAP after exclusion of readily apparent causes by history, routine laboratory tests, and conventional imaging, not necessarily including MRCP, EUS (transesophageal endoscopic ultrasound), ERCP with or without manometry, or genetic testing (grade 1B, 75% probably or definitely agree with the definition) [41]. For 70–80% of patients with RAP, a specific cause may be identified [42] such as gallstone disease (especially biliary microlithiasis), excessive alcohol consumption, hypertriglyceridemia, medications, intraductal papillary mucinous neoplasms (IPMNs), genetic mutations, hypercalcemia, sphincter of Oddi dysfunction, and pancreatobiliary ductal anomalies. Apart from laboratory and imaging tests to identify causes of acute pancreatitis, the investigation of RAP requires a detailed assessment of pancreatic ductal anatomy. MRCP enables the evaluation of ductal anatomy, to identify parenchymal abnormalities including cystic lesions and may be the best available test to diagnose non-calcific chronic pancreatitis; it should be considered a key component in the evaluation of RAP [43]. MRCP can be performed also after the intravenous injection of secretin, that stimulates water and bicarbonate secretions from the exocrine cells of the pancreas leading to more conspicuous visualization of main pancreatic duct and diseased side branches. Secretin-enhanced MRCP is useful in showing potentially treatable causes of RAP, such as divisum anatomy, ductal strictures and gives an assessment of the exocrine function of the gland [44]. A study comparing patients with RAP to community control subjects showed a higher frequency of divisum in patients with RAP [45]. It is clinical practice to investigate for anomalous ductal anatomy in patients with recurrent acute pancreatitis, indeed secretin-enhanced MRCP (S-MRCP) can reveal morphologic features of ductal anatomy like a cystic dilatation of the end of the Santorinicele, multiple dilated side branches, or subclinical ductal changes of chronic pancreatitis, obviating the need for ERCP. It has been seen also that functional deficiencies occur in about one-third of patients with RAP, even when no anatomic ductal disease is found, adding to S-MRCP the potential to quantify pancreatic exocrine reserve. S-MRCP has a higher sensitivity and specificity (86%) compared with routine MRCP (57%) for diagnosing divisum, and it is superior also in diagnosing main duct dilation, 86% versus 42% [45]. The most impressive added benefit of se-cretin-enhanced MRCP is the detection of side branch dilation, suggesting the superior of S-MRCP to conventional MRCP for the diagnosis of early chronic pancreatitis. By the way it is important to underline that secretin is an expensive drug.

## Conclusion

AP is an evolving condition, whose severity may change during the disease. In the early phase, during the first week after onset, the disease manifests as a systemic inflammatory response; at this time clinical severity and treatment are mainly determined based on type and degree of organ failure. In the late phase, from the second week to months, imaging plays an important role in the diagnosis and staging. Wider availability and good image quality make CT the mostly used imaging technique. In a good report the radiologists must define the type of AP and its complications; it should include a statement about the presence or absence of necrosis about the location and the amount of necrotic gland. Also, local complication should be described in the terms of location, size, appearance, and presence or absence of a mature wall. The collection should be named according to the revised Atlanta classification lexicon, and the impression section should contain a summary of the findings in order to standardize treatment, communicate imaging appearance using common terminology, and hopefully improve patient outcomes.

## Data Availability

Data sharing is not applicable to this article as no datasets were generated or analyzed.
